# Myelination of the Postnatal Mouse Cochlear Nerve at the Peripheral-Central Nervous System Transitional Zone

**DOI:** 10.3389/fped.2013.00043

**Published:** 2013-12-17

**Authors:** Jue Wang, Baofu Zhang, Hui Jiang, Lei Zhang, Danzheng Liu, Xiao Xiao, Hannah Ma, Xuemei Luo, Dennis Bojrab, Zhengqing Hu

**Affiliations:** ^1^Department of Otolaryngology-HNS, Wayne State University School of Medicine, Detroit, MI, USA; ^2^Department of Otolaryngology, Jinshan Hospital, Fudan University, Shanghai, China; ^3^Department of Otolaryngology, Zhongshan Hospital, Fudan University, Shanghai, China

**Keywords:** myelin, PNS-CNS transitional zone, cochlear nerve, oligodendrocyte, Schwann cell

## Abstract

In the nerve roots of vertebrates, the peripheral nervous system (PNS) and central nervous system (CNS) interface at the PNS-CNS transitional zone (PCTZ), which consists of cell boundaries with various myelin components. We have recently shown that the mouse cochlear nerve presents an exceptionally long segment of the CNS tissue extending into the PNS using light microscopy. However, it is unclear how oligodendrocytes and Schwann cells contribute to the formation of myelin components of the PCTZ. It is undetermined how myelination is initiated along the cochlear nerve, and when it adopts a mature pattern. In this study, immunofluorescence using antibodies specific to oligodendrocyte marker myelin oligodendrocyte glycoprotein (MOG) and Schwann cell marker myelin protein zero (MPZ) were used to detail the expression of myelin components along the postnatal mouse cochlear nerve. We found that the expression of MPZ was initially observed in the soma of bipolar spiral ganglion neurons at postnatal day 0 (P0) and progressed to the central and peripheral processes after P8–P10. Myelination of the CNS tissue was initiated in close proximity to the PCTZ from P7 to P8 and then extended centrally. Myelination of the PCTZ reached a mature style at P14, when the interface of the expression of MOG and MPZ was clearly identified along the cochlear nerve. This knowledge of PCTZ formation of the cochlear nerve will be essential to future myelination research, and it will also gain clinical interest because of its relevance to the degeneration and regeneration of the auditory pathway in hearing impairment.

## Introduction

In the nervous system of vertebrates, the peripheral nervous system (PNS) and central nervous system (CNS) transitional zone (PCTZ) constitutes the boundary of the CNS and PNS. The PCTZ of cranial nerves, such as oculomotor, trochlear, and abducent nerves, is usually dome shaped with the CNS compartment extending peripherally along the nerve trunks (1, 2). In most nerve roots, the PCTZ is located within a few millimeters from the CNS surface. The nerve fibers usually continue at the PCTZ, while myelin components of the CNS and PNS interface to form the PCTZ (3, 4). Oligodendrocytes and Schwann cells are the myelinating glia of the CNS and PNS respectively, which form unique heminodes at the PCTZ (5, 6). During development, oligodendrocytes originate from the neural tube and Schwann cells are derived from the neural crest, which contribute to the distinct properties of their morphology and molecular components (7). For example, myelin protein zero (MPZ) is specifically expressed in Schwann cells while myelin oligodendrocyte glycoprotein (MOG) is detected in oligodendrocytes (4).

Cochlear nerve is responsible for auditory signals transferring from the cochlea to the cochlear nucleus (CN). A light microscopy study suggested that the rat cochlear nerve presented an exceptionally long segment of CNS tissue extending into the PNS, and the PCTZ located within the internal acoustic meatus (8, 9). Our recent light microscopy study in the mouse model confirmed this observation. In addition, we found that the PCTZ of the mouse cochlear nerve was located within the modiolus at the basal cochlear turn level (10).

In a previous study using antibodies specific to PNS and CNS myelin, it was observed that peripheral myelin proteins were specifically expressed in the segment between the perikaryon of spiral ganglion neurons and the PCTZ along the cochlear nerve, while the central type myelin was expressed between the PCTZ and the brainstem. However, it is unclear how oligodendrocytes and Schwann cells contribute to the formation of myelin components along the cochlear nerve. It remains unknown how myelination is initiated along the cochlear nerve, and when it adopts a mature pattern. In this study, we harvested the postnatal mouse cochlear nerve and used immunofluorescence analyses to address these questions.

## Materials and Methods

### Animal and groups

All animal procedures were approved by local Institutional Animal Care and Use Committee (IACUC). Swiss Webster mice were used in this study at the following ages: postnatal day 0 (P0, the day of birth), 1, 3, 5, 7, 8, 10, 14, and 30 of either sex. The tissues from P0 mice were harvested at approximately 3–4 h after birth. Each age point comprised of five replicates.

### Tissue harvest and sample preparation

A novel cochlea-cochlear nerve-CN cryosection model was used in this study (10). P0–P14 mice were euthanized with decapitation, and P30 mice were euthanized with an overdose of CO_2_. The inner ear, brainstem, and the connecting cochlear nerve were identified and fixed in 4% paraformaldehyde (Sigma) overnight at 4°C. The specimens from P7 to P30 were treated with 0.1 M EDTA (Sigma) for approximately 5 days for decalcification. The samples were incubated in 30% sucrose (Sigma) for cryoprotection followed by cryosectioned on a cryostat (Leica) at a thickness of approximately 10 μm. The sectioning was started on the surface of round window and parallel to the modiolus. The sections containing the entire cochlea, the cochlear nerve, and the CN were collected for the following immunofluorescence study.

### Immunofluorescence study

Cryosections were incubated in PBS containing 5% normal donkey serum (Jackson ImmunoResearch) and 0.2% Triton X-100 (Sigma) for 30 min, and then in the primary antibodies overnight at 4°C. The primary antibodies used in this study included monoclonal mouse anti-MOG (1:100, Millipore) and chicken anti-MPZ (1:200, Millipore). Dylight conjugated secondary antibodies (Jackson ImmunoResearch) were applied to cryosections for 2 h at room temperature. The samples were observed using an epifluorescence microscopy (Leica) or confocal microscopy (Leica).

## Results

### Expression of schwann cell protein MPZ

**Figure 1:**
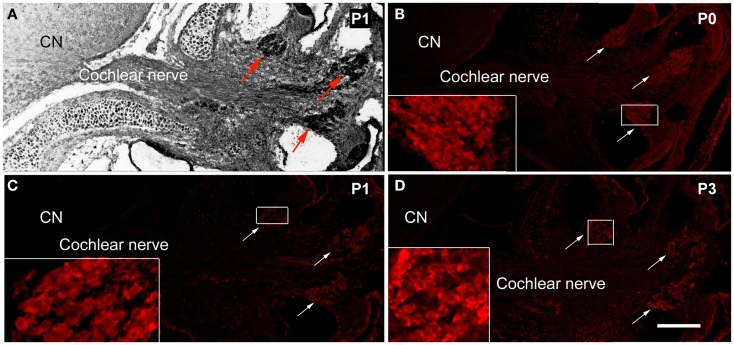
**The expression of MPZ in the inner ear of postnatal mouse**. **(A)** A light microscopy image showed the anatomy of postnatal day 1 (P1) mouse cochlea, spiral ganglion region (arrows), cochlear nerve, and cochlear nucleus (CN). The myelination of the cochlear nerve distal to the PCTZ, which was determined by immunostaining of MPZ [red, arrows in **(B–D)**], was found around the soma of the spiral ganglion neurons in P0 **(B)**, P1 **(C)**, and P3 **(D)** mice. Scale: 50 μm shown in **(D)**.

**Figure 2:**
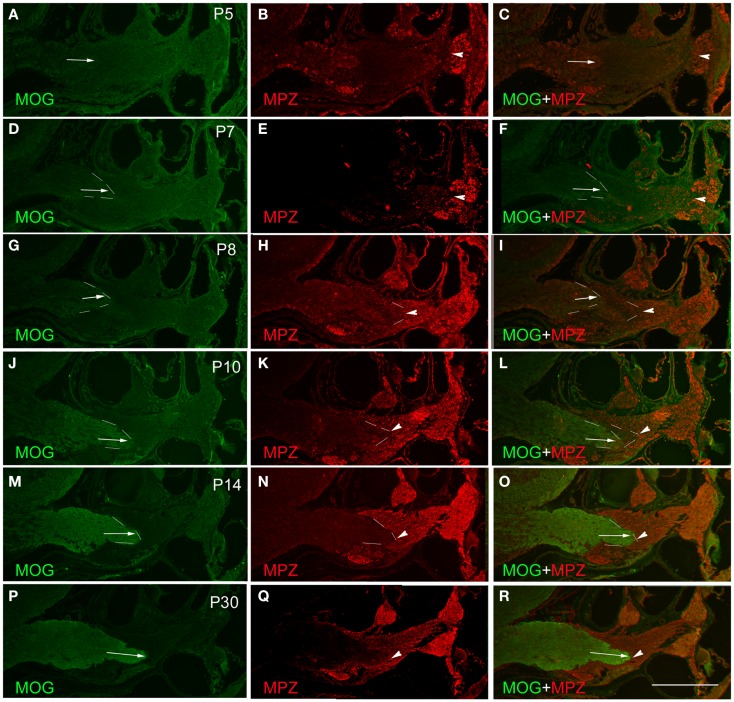
**Determination of PCTZ formation along the postnatal mouse cochlear nerve using MOG and MPZ immunostaining**. The expression of MOG was weak and hardly detectable during postnatal days 5–7 (P5–P7) [arrows in **(A)**, **(C)**, **(D)**, and **(F)**]. From P8 to P10, the expression of MOG was increased and located at the level of basal cochlear turn [arrows in **(G)**, **(I)**, **(J)**, and **(L)**; dotted lines indicate the boundary]. The expression of MOG was strong and obtained the mature pattern at P14–P30 [arrows in **(M)**, **(O)**, **(P)**, and **(R)**]. The expression of MPZ around the spiral ganglion region was weak on P5 **(B)**, strengthened from P7 to P10 [arrowheads in **(E)**, **(H)**, and **(K)**; dotted lines indicate the boundary], and held stable after P14 [arrowheads in **(N)** and **(Q)**; dotted lines indicate the boundary]. MPZ expression was initially observed along the cochlear nerve in close proximity to the apical turn of the cochlea as early as P5 [arrowhead in **(B)**]. Its expression extended centrally toward PCTZ from P7 to P10 [arrowheads in **(E)**, **(F)**, **(H)**, **(I)**, **(K)**, and **(L)**], and reached the PCTZ after P14 [arrowheads in **(N)**, **(O)**, **(Q)**, and **(R)**]. Scale: 50 μm shown in **(R)**.

The expression of PNS Schwann cell protein MPZ was detected at the spiral ganglion area from P0 (Figure 1). During P0–P3, the expression of MPZ was at very low levels and restricted to the soma region of spiral ganglion neurons (Figures 1B,D). During P5–P7, the expression level of MPZ was increased but still confined to the soma area of spiral ganglion neurons (Figures 2B,E). From P8 to P10, the expression of MPZ was observed at the peripheral processes to the organ of Corti and the central processes to the CN (Figures 2H,K). The expression of MPZ reached a mature pattern from P14, which was strong around the soma, the peripheral and central processes of spiral ganglion neurons (Figure 2N). At P30, MPZ immunostaining was stably observed at spiral ganglion neurons, including the soma area, the peripheral and central processes (Figure 2Q).

### Expression of oligodendrocyte protein MOG

The expression of the CNS oligodendrocyte protein MOG was hardly detectable before P5 (Figures 2A,C). MOG expression was weak on P7 (Figures 2D,F), gradually became stronger from P8 to P14 (Figures 2G,J,M), and stayed consistent after P14 (Figure 2P). The expression of MOG in the CNS portion was initially observed at the distal part of the central projections that was close to the PCTZ at P7–P8 (Figures 2D,G). During P10–P14, MOG expression extended centrally toward the CN (Figures 2G,J,M,P) and obtained a mature pattern after P14.

### Determination of PCTZ using oligodendrocyte and schwann cell markers

Using antibodies for oligodendrocytes and Schwann cells, the interface of PCTZ was hard to observe before P7 because the low expression level of MOG and MPZ. At P8, the expression of MOG and MPZ was increased but a gap existed along the cochlear nerve between the portions stained with MOG and MPZ (Figures 2G–I). At P10, the area labeled with MOG and MPZ was getting closer but did not form an apparent boundary along the cochlear nerve (Figures 2J–L). At P14, the expression of MOG and MPZ was strong and formed a distinct boundary along the cochlear nerve (Figures 2M,O). At P30, the PCTZ interface was identified by the boundary interfaced with the expression of MOG and MPZ (Figures 2P–R).

## Discussion

In this study, we investigated the formation of myelin components along the postnatal mouse cochlear nerve using antibodies specific for the myelin of the PNS and CNS. We found that MPZ was detected in the soma region of spiral ganglion neurons as early as P0, while the peripheral and central processes of spiral ganglion neurons were labeled by MPZ from P8 to P10. Myelination of the CNS tissue was initiated from P7 to P8 and located in close proximity to the PCTZ region. The formation of the PCTZ and myelination of the mouse cochlear nerve reached a mature pattern after P14.

In a previous study using a rat cochlear nerve model, no MPZ immunoreaction was observed at P0. On P8, many spiral ganglion neurons appeared to be surrounded by MPZ immunostaining (9). In our mouse model, myelination of the PNS portion of the cochlear nerve, which was formed by Schwann cells, was observed around the soma of spiral ganglion neurons as early as P0. In this study, it was also observed that myelination by Schwann cells along the peripheral and central processes of the spiral ganglion neurons began around P8–P10 and then extended centrally toward the PCTZ. After P14, myelination of PNS portion of the mouse cochlear nerve reached a mature state. The reason for this different MPZ expression pattern between the rat and mouse models is obscure. It may be related to the species difference, which requires further investigation.

Our previous study has shown that the CNS tissue extended an exceptionally long distance along the cochlear nerves into the inner ear in the rat and mouse models (10). We found that the mouse PCTZ migrated peripherally from P0 to P5, from the sub-arachnoid segment of the cochlear nerve to the internal auditory meatus, and then toward the basal cochlear turn to reach a mature pattern after P7 (10). In this study, we found that myelination of the CNS portion of the mouse cochlear nerve was initiated on P7–P8, which was indicated by the MOG expression along the central portion of the cochlear nerve. Myelination of oligodendrocytes was initiated in close proximity to the PCTZ, then extended centrally to the CN, and obtained a mature pattern after P14. These results revealed that the acquisition of mature CNS tissue projection pattern observed in the light microscopy is consistent with myelination of the central portion of the cochlear nerve using MOG immunostaining.

In summary, we found that the expression of MPZ was detected around the soma of spiral ganglion neurons as early as P0 and at the peripheral and central processes during P8–P10. Myelination of CNS projections, which was indicated by MOG immunostaining, was initiated in close proximity to the PCTZ at P7–P8. Understanding of cochlear nerve myelination around the PCTZ will be helpful for future myelination studies at the molecular levels. Additionally, the knowledge of myelination along the cochlear nerve has clinical significance owing to its relevance to cochlear nerve disorders in hearing loss, neurofibromatosis type II, head injuries, and other inner ear disorders. Moreover, advances in stem cell technology provide novel opportunities for auditory pathway regeneration (11, 12). The information on cochlear nerve myelination and PCTZ formation will therefore assist in developing novel treatment options for auditory pathway regeneration using stem cell-based strategies.

## Authors Contribution

Jue Wang, Baofu Zhang, and Hui Jiang conduct the experiment, collected the data, revise the manuscript, and approve the final version of the paper. Lei Zhang, Danzheng Liu, Xiao Xiao, Hannah Ma, Xuemei Luo, Dennis Bojrab II, and Zhengqing Hu contribute to design the experiment, analyze the data, write and revise the manuscript, and approve the final version of the paper.

## Conflict of Interest Statement

The authors declare that the research was conducted in the absence of any commercial or financial relationships that could be construed as a potential conflict of interest. The Associate Editor J.M. Coticchia and Review Editor Y. Zhu declares that, despite being affiliated to the same institution as all authors, the review process was handled objectively and no conflict of interest exists.
